# Diagnostic Performance of First-Trimester Placental Shear Wave Elastography for Predicting Hypertensive Disorders of Pregnancy During the 11- to 14-Week Scan in a Tertiary Care Hospital

**DOI:** 10.7759/cureus.113098

**Published:** 2026-07-21

**Authors:** Rewati R Pokhrel, Barun K Sharma, Pesona G Lucksom, Tsella Lachungpa, Abhinash Sharma, Neeraj Rai, Karma T Bhutia, Shubham Kumar, Akansha Rana, Kartikeya Upadhyay

**Affiliations:** 1 Radiodiagnosis, Central Referral Hospital, Gangtok, IND; 2 Radiodiagnosis, Sikkim Manipal Institute of Medical Sciences, Gangtok, IND; 3 Obstetrics and Gynecology, Sikkim Manipal Institute of Medical Sciences, Gangtok, IND

**Keywords:** emax average, first trimester, hypertensive disorders of pregnancy, placental shear wave elastography, placental stiffness, preeclampsia, pregnancy-induced hypertension, ultrasonography

## Abstract

Introduction

Hypertensive disorders of pregnancy (HDP) are important causes of maternal and perinatal morbidity. Although clinical hypertension usually becomes evident after 20 weeks of gestation, abnormal placentation begins much earlier. Placental shear wave elastography (SWE) is a quantitative ultrasound technique that measures tissue stiffness and may help identify early biomechanical changes in the placenta before clinical disease becomes apparent. This study evaluated the association and diagnostic performance of placental SWE performed during the 11- to 14-week scan for predicting the later development of HDP.

Methods

This hospital-based prospective analytical observational study was conducted in the Department of Radiodiagnosis, Central Referral Hospital, Sikkim Manipal Institute of Medical Sciences, Gangtok, Sikkim. Pregnant women attending routine first-trimester nuchal translucency and nasal bone (NTNB) scans were enrolled after informed consent. Placental SWE was performed using a Mindray DC-80 X Insight ultrasound system (Shenzhen Mindray Bio-Medical Electronics Co., Ltd., Shenzhen, China) with an SC5-1E curvilinear probe. Three approximately 5 mm regions of interest were placed within the placental parenchyma, avoiding placental margins, cord insertion, placental lakes, focal heterogeneity, and excessive probe pressure. Averaged EMAX, EMEAN, and EMIN values were used for analysis. Women were followed at 28, 32, and 36 weeks and at 40 weeks or delivery. Group comparison and receiver operating characteristic (ROC) curve analysis were performed.

Results

Of 80 enrolled women, five were excluded due to loss to follow-up or abortion. The final analysis included 75 women. Among these women, 57 remained normotensive, and 18 developed HDP. The HDP group included seven women with pregnancy-induced hypertension (PIH)/gestational hypertension and 11 with preeclampsia. No case of eclampsia was recorded. EMAX Average was significantly higher in the HDP group than in the non-HDP/normotensive group (13.09 ± 4.79 kPa vs 10.24 ± 4.02 kPa; p = 0.015). EMEAN Average was also significantly higher (7.33 ± 2.88 kPa vs 5.93 ± 2.07 kPa; p = 0.027), while EMIN Average did not show a statistically significant difference (4.11 ± 1.62 kPa vs 3.47 ± 1.38 kPa; p = 0.105). On ROC analysis, EMAX Average showed the best diagnostic performance, with an area under the curve of 0.682 (95% CI: 0.564-0.785; p = 0.009). At a cut-off value of >11.71 kPa, sensitivity was 61.1%, and specificity was 73.7%. EMEAN Average showed an area under the curve of 0.643 (p = 0.058), while EMIN Average showed an area under the curve of 0.607 (p = 0.157).

Conclusion

First-trimester placental SWE was feasible during the routine 11- to 14-week ultrasound scan. EMAX Average showed the best diagnostic performance for later HDP, while EMEAN Average showed a significant group difference but did not reach statistical significance on ROC analysis. Overall diagnostic performance was moderate. Placental SWE should therefore be considered a complementary imaging biomarker rather than a standalone screening test for HDP.

## Introduction

Hypertensive disorders of pregnancy (HDP) are major contributors to maternal and perinatal morbidity and mortality [1-3]. Globally, preeclampsia affects approximately 3%-8% of women who give birth, and hypertensive disorders are responsible for around 16% of maternal deaths worldwide, equivalent to approximately 42,000 maternal deaths in 2023 [4]. In India, a systematic review and meta-analysis estimated the pooled prevalence of HDP to be approximately 11% [5]. HDP includes pregnancy-induced hypertension (PIH), preeclampsia, and eclampsia, and is associated with adverse outcomes such as fetal growth restriction, placental abruption, perinatal mortality, and preterm birth [1-3]. PIH is defined as new-onset hypertension after 20 weeks of gestation in a previously normotensive woman without proteinuria or systemic features. Preeclampsia is a multisystem disorder characterized by hypertension with proteinuria and/or maternal end-organ dysfunction or uteroplacental dysfunction [3].

The clinical manifestations usually appear in the second half of pregnancy; however, the underlying pathological process often begins much earlier. Abnormal placentation, defective trophoblastic invasion, and incomplete spiral artery remodeling are central mechanisms in preeclampsia and related hypertensive disorders [6-8]. In a normal pregnancy, maternal spiral arteries are transformed into low-resistance vascular channels that support adequate uteroplacental perfusion. When this process is defective, placental hypoperfusion, oxidative stress, and maternal endothelial dysfunction may occur [7,9].

Current first-trimester screening strategies use combinations of maternal risk factors, blood pressure or mean arterial pressure, uterine artery Doppler, and biochemical markers such as placental growth factor and pregnancy-associated plasma protein-A [10,11]. These combined approaches are useful, but no single marker can reliably identify all women who will later develop HDP. Additional imaging biomarkers that assess placental structure or function may therefore be useful.

Ultrasound elastography is a non-invasive technique that assesses tissue stiffness. Shear wave elastography (SWE) uses acoustic radiation force to generate shear waves within tissue and measures their propagation velocity. Stiffer tissues allow faster shear wave propagation, and the values can be expressed in kilopascals or meters per second [12,13]. In placental imaging, SWE may provide quantitative information about tissue biomechanics that is not available from conventional grayscale ultrasound or Doppler assessment alone.

Placental stiffness may be altered by malperfusion, ischemia, fibrin deposition, stromal changes, and villous abnormalities. Previous studies have reported increased placental stiffness in pregnancies complicated by preeclampsia or placental dysfunction [14-18]. However, previous studies have varied in gestational age, ultrasound platform, placental sampling technique, and reported elastographic parameter, which limits direct comparison between studies. Moreover, many studies have been performed in the second or third trimester, when clinical disease may already be present. First-trimester placental SWE remains less extensively studied, particularly in hospital-based cohorts from North-East India.

The present study was conducted to evaluate the association and diagnostic performance of placental SWE performed during the 11- to 14-week scan for the later development of HDP. The HDP outcome included PIH/gestational hypertension, preeclampsia, and eclampsia. The elastographic parameters assessed were EMAX Average, EMEAN Average, and EMIN Average. The study also aimed to determine their diagnostic performance and exploratory optimal cut-off values.

## Materials and methods

Study design and setting

This was a hospital-based prospective analytical observational study conducted in the Department of Radiodiagnosis, Central Referral Hospital, Sikkim Manipal Institute of Medical Sciences (SMIMS), Gangtok, Sikkim, India. The study was carried out among pregnant women attending routine first-trimester nuchal translucency and nasal bone (NTNB) scan.

Ethical considerations

The study was conducted after approval from the Institutional Research Committee, SMIMS (IRC Registration No.: IRC/2024-58; dated 02.02.2024), and the Institutional Ethics Committee, SMIMS (IEC Registration No.: SMIMS/IEC/2024-30; dated 01.03.2024). Written informed consent was obtained from all participants before enrolment. Participation was voluntary. No additional invasive procedure, drug administration, contrast administration, or research-related blood withdrawal was performed. Ultrasound examination was performed following the ALARA principle, with avoidance of unnecessary repeated acquisitions and excessive probe pressure.

Study population

Pregnant women aged more than 18 years with a live singleton pregnancy between 11 and 14 weeks of gestation who were attending routine NTNB ultrasonography and were available for follow-up were included. Women with multiple pregnancy, known chronic hypertension or hypertension before pregnancy, hypertension detected before 20 weeks, known renal disease or autoimmune disorder likely to confound outcome assessment, fetal congenital anomaly at the initial scan, unsuitable placental location for reliable elastography, inadequate image quality, or unavailable follow-up were excluded.

Sample size

The sample size was calculated for receiver operating characteristic (ROC) curve analysis. Based on the first-trimester placental SWE study by Sirinoglu et al., the expected area under the ROC curve was assumed to be approximately 0.82 [18]. Considering 80% power and a 10% error level, the minimum required sample size was calculated to be 79 participants. To account for possible attrition, 80 pregnant women were initially enrolled.

Ultrasound and elastography protocol

All ultrasound examinations were performed using the Mindray DC-80 X Insight ultrasound system (Shenzhen Mindray Bio-Medical Electronics Co., Ltd., Shenzhen, China) equipped with SWE software. An SC5-1E curvilinear probe with a frequency range of 1-5 MHz was used. Women were examined in the supine position. Routine first-trimester ultrasound assessment was performed first, followed by placental SWE during the same visit.

B-mode and SWE images were displayed simultaneously using split-screen imaging. Adequate coupling gel was used, and excessive transducer pressure was avoided. Maternal breathing artifact and fetal movement were avoided as far as possible during acquisition. Placental location and placental depth were recorded during the ultrasound assessment. Placental margins, cord insertion site, placental lakes, large vascular structures, focal heterogeneity, and the myometrial interface were avoided during region-of-interest placement.

Placental SWE examinations were performed by four radiologists with experience ranging from 2 to 17 years, from Senior Resident to Professor level. All operators were familiar with obstetric ultrasound and followed the same predefined SWE acquisition protocol. The operators performing SWE were blinded to maternal risk factors and subsequent pregnancy outcome at the time of examination. SWE measurements were accepted only when the machine-generated Motion Stability Index (M-STB) showed five stars, indicating adequate measurement stability. Measurements affected by fetal movement, maternal breathing artifact, excessive probe pressure, poor elastographic filling, or inadequate stability were avoided and reacquired where necessary.

Three regions of interest were placed within the placental parenchyma. Each region of interest measured approximately 5 mm in diameter. Measurements were obtained from different representative placental parenchymal regions where technically feasible. For each region of interest, EMAX, EMEAN, and EMIN were recorded in kilopascals. For final analysis, the average of the three measurements was used for each parameter. The final analyzed variables were EMAX Average, EMEAN Average, and EMIN Average.

Follow-up and outcome definition

All enrolled women were followed prospectively during pregnancy. Blood pressure records and clinical follow-up details were reviewed at 28, 32, and 36 weeks and at 40 weeks or delivery, depending on the timing of delivery and availability of records. In women who delivered before 40 weeks, the delivery record and the latest available antenatal blood pressure record were used.

The primary outcome was the development of HDP after 20 weeks of gestation. PIH, also referred to as gestational hypertension, was defined as a previously normotensive woman developing new-onset systolic blood pressure ≥140 mmHg and/or diastolic blood pressure ≥90 mmHg after 20 weeks, without proteinuria or features of preeclampsia. Preeclampsia was defined as new-onset hypertension after 20 weeks associated with proteinuria and/or maternal end-organ dysfunction or uteroplacental dysfunction. Eclampsia was defined as convulsions in a woman with preeclampsia, in the absence of another identifiable neurological cause [3].

Outcome assessment was based on antenatal and delivery clinical records. The clinicians assessing pregnancy outcome were blinded to the first-trimester placental SWE values. Proteinuria, maternal end-organ dysfunction, and uteroplacental dysfunction were assessed from clinical records according to the predefined study criteria.

For final analysis, women were categorized into two groups: non-HDP/normotensive and HDP. The HDP group included women who developed PIH/gestational hypertension, preeclampsia, and eclampsia.

Statistical analysis

Data were entered into Microsoft Excel (Microsoft® Corp., Redmond, WA, USA) and analyzed using IBM SPSS Statistics for Windows, Version 21 (Released 2012; IBM Corp., Armonk, NY, USA). Categorical variables were expressed as frequency and percentage, and continuous variables were expressed as mean ± standard deviation. Continuous variables between the HDP and non-HDP groups were compared using the independent sample t-test when distributional assumptions were fulfilled. A p-value <0.05 was considered statistically significant.

ROC curve analysis was performed to assess the diagnostic performance of EMAX Average, EMEAN Average, and EMIN Average for the prediction of HDP. The area under the ROC curve was calculated for each averaged elastography parameter. Optimal cut-off values were derived using the Youden index. Sensitivity, specificity, positive likelihood ratio, negative likelihood ratio, positive predictive value, and negative predictive value were calculated for selected cut-off values. Pairwise comparison of ROC curves was also performed.

Multivariable logistic regression was not performed because only 18 HDP outcome events occurred in the final analyzed cohort. Inclusion of multiple predictors in this setting would have increased the risk of model overfitting and unstable estimates. Therefore, the analysis was interpreted as a univariable group comparison and an exploratory diagnostic-performance assessment rather than as proof of independent predictive ability.

**Figure 1:**
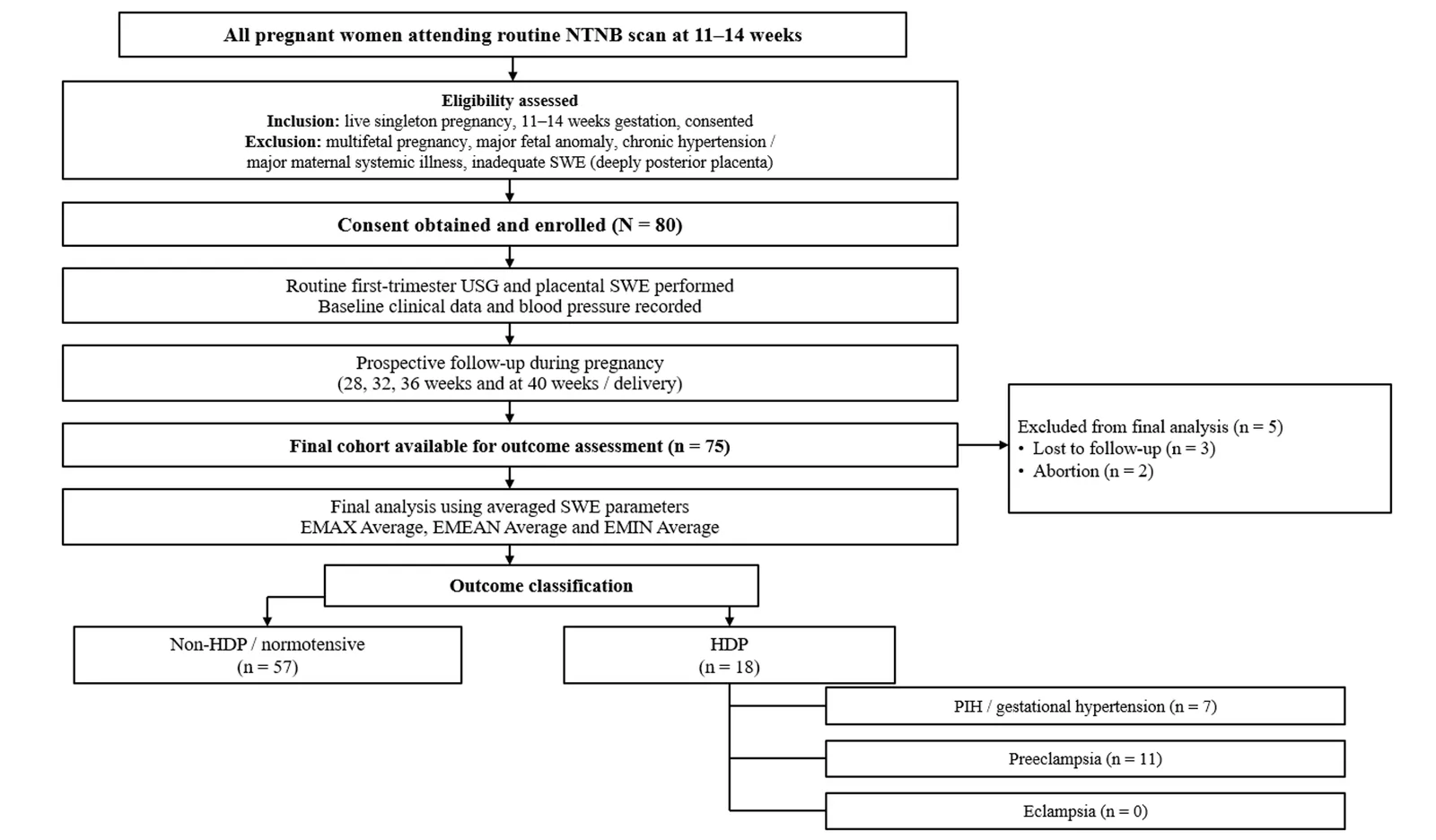
Study flowchart showing participant selection, consent, ultrasound elastography acquisition, follow-up, and final outcome assessment. NTNB: nuchal translucency and nasal bone scan; SWE: shear wave elastography; HDP: Hypertensive disorders of pregnancy; PIH: pregnancy-induced hypertension

Figure 1 summarizes the study from participation to final outcome assessment. 

## Results

Study cohort and baseline profile

A total of 80 pregnant women attending routine first-trimester NTNB scan were initially enrolled. Three women were lost to follow-up, and two cases resulted in abortion. These five cases were excluded from the final analysis. The final analyzed cohort included 75 women with complete follow-up data.

The baseline maternal and obstetric profile of the final analyzed cohort is presented in Table 1. The mean maternal age was 29.69 ± 4.09 years, and the mean ultrasound-based average ultrasound age (USG-AUA)-based gestational age at the time of the NTNB scan was 12.59 ± 0.87 weeks. G1 and P0 were the most frequent gravida and parity categories, respectively.

**Table 1:** Baseline maternal and obstetric profile of the final analyzed cohort. Values are expressed as mean ± standard deviation, range, or number and percentage, as applicable. USG-AUA: ultrasound-based average ultrasound age; G: gravida; P: parity

Variable (n = 75)	Value / n (%)	Range
Maternal age (years)	29.69 ± 4.09	19-39
USG-AUA-based gestational age (weeks)	12.59 ± 0.87	11.00-14.00
Gravida G1	39 (52.0%)	-
Gravida G2	28 (37.3%)	-
Gravida G3	6 (8.0%)	-
Gravida G4	2 (2.7%)	-
Parity P0	40 (53.3%)	-
Parity P1	30 (40.0%)	-
Parity P2	4 (5.3%)	-
Parity P3	1 (1.3%)	-

**Figure 2:**
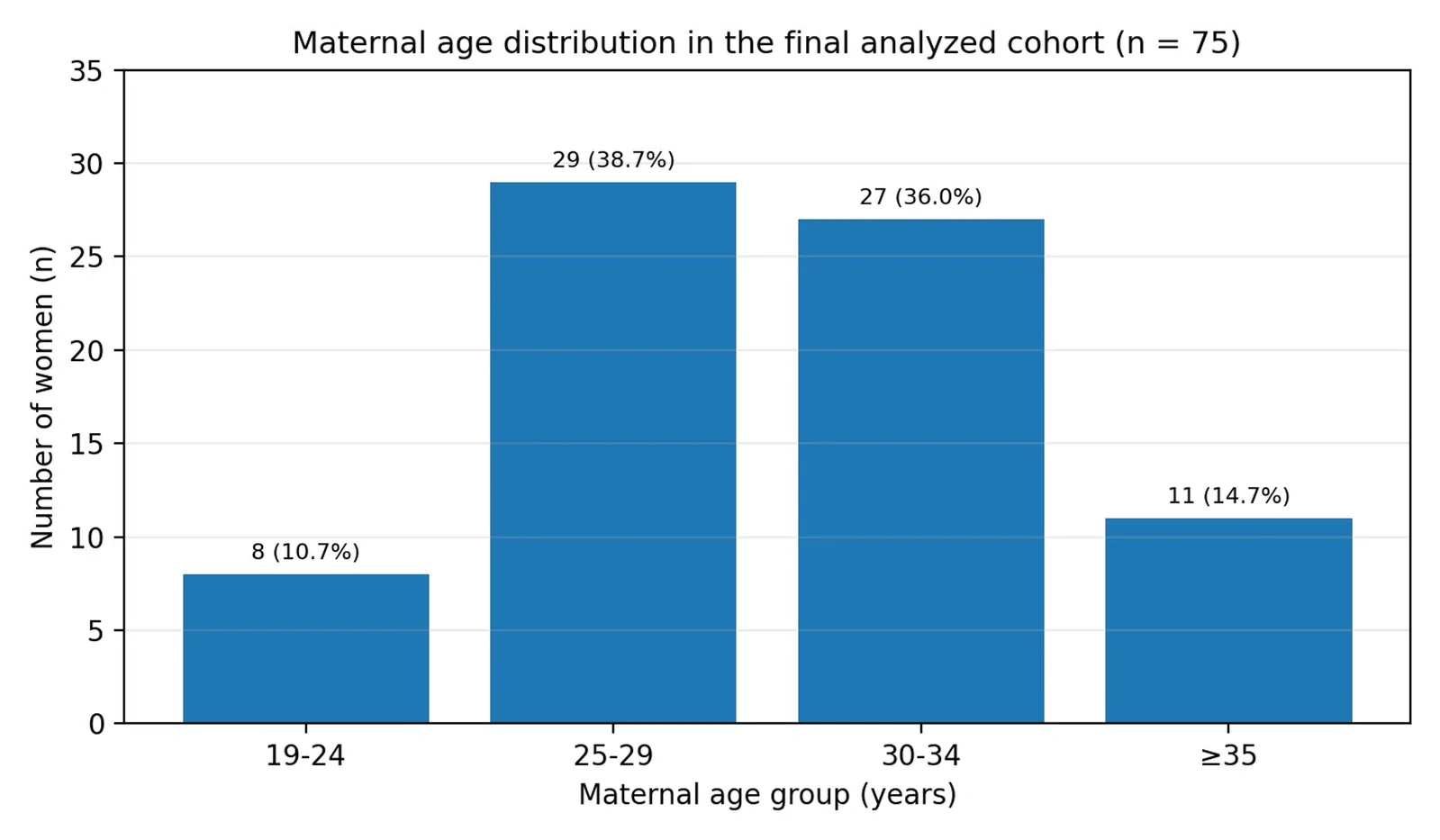
Bar chart showing maternal age distribution in the final analyzed cohort.

**Figure 3:**
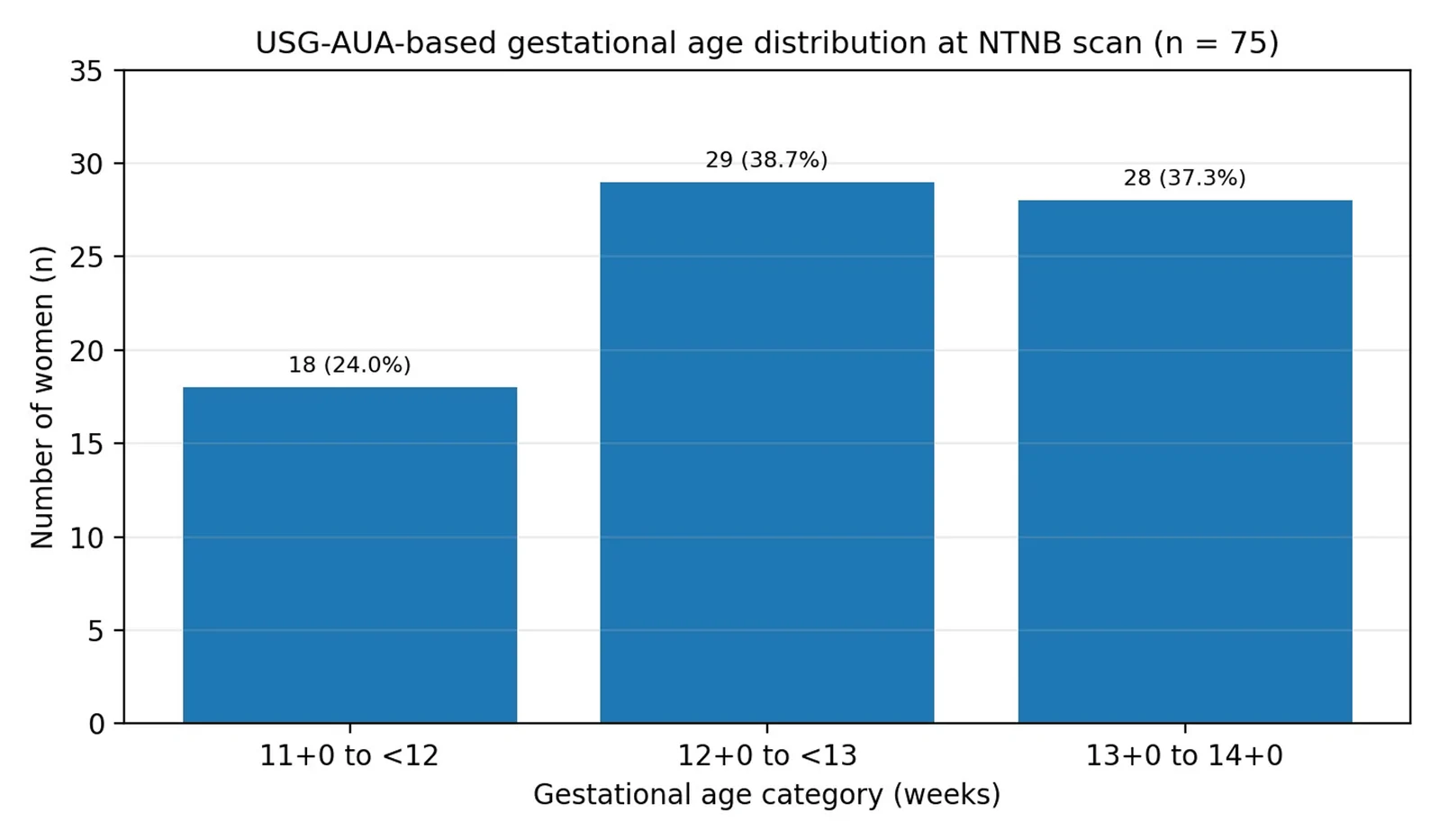
Bar chart showing USG-AUA-based gestational age distribution at the time of NTNB scan. USG-AUA: ultrasound-based average ultrasound age; NTNB: nuchal translucency and nasal bone scan

The age distribution of the final analyzed cohort is shown in Figure 2. Most women were in the 25-29 years age group, followed by the 30-34 years age group. The USG-AUA-based gestational age distribution at the time of the NTNB scan is shown in Figure 3. All women were scanned within the 11+0 to 14+0 weeks window, with the largest proportion scanned between 12+0 and <13 weeks.

Outcome distribution

**Figure 4:**
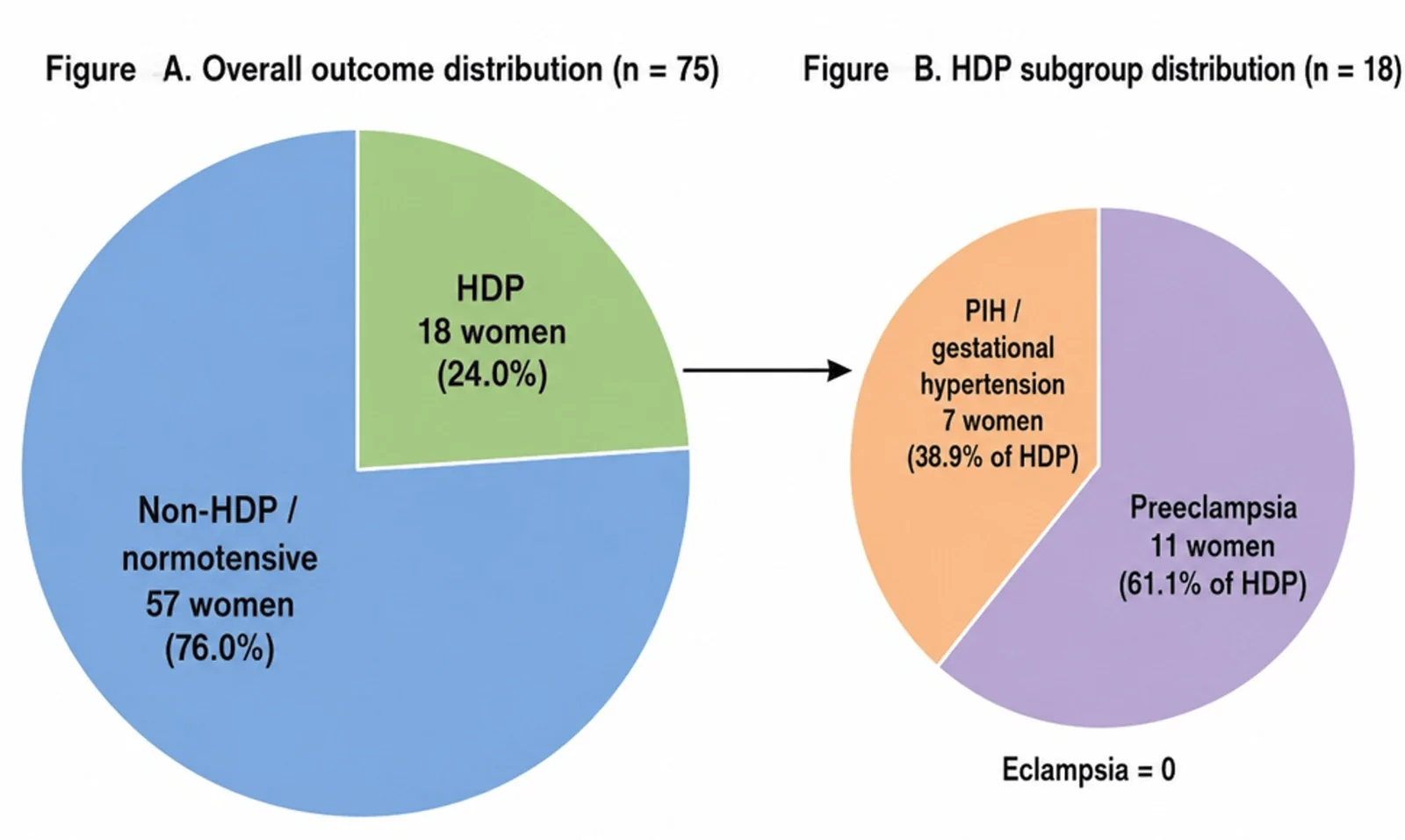
Pie charts showing outcome distribution in the final analyzed cohort. Figure A shows the overall outcome distribution, with 57 of 75 women (76.0%) remaining normotensive and 18 of 75 women (24.0%) developing HDP. Figure B shows the distribution within the HDP group, including PIH/gestational hypertension in 7 of 18 women (38.9%) and preeclampsia in 11 of 18 women (61.1%). No woman developed eclampsia. HDP: hypertensive disorders of pregnancy; PIH: pregnancy-induced hypertension

Among the 75 women included in the final analysis, 57 remained normotensive during pregnancy and 18 developed HDP. The HDP group included seven women with PIH/gestational hypertension and 11 women with preeclampsia. No case of eclampsia was recorded. The outcome distribution is shown in Figure 4.

Average placental SWE values

For each woman, three placental SWE measurements were obtained, and averaged values were used for final analysis. In the final cohort, the mean EMAX Average was 10.92 ± 4.36 kPa, the mean EMEAN Average was 6.26 ± 2.35 kPa, and the mean EMIN Average was 3.62 ± 1.46 kPa.

Comparison between HDP and non-HDP groups

Average placental SWE parameters were compared between the HDP and non-HDP groups using an independent sample t-test, as shown in Table 2. EMAX Average and EMEAN Average were significantly higher in the HDP group, while EMIN Average did not show a statistically significant difference.

**Table 2:** Comparison of average placental SWE parameters between HDP and non-HDP groups. HDP: hypertensive disorders of pregnancy, SWE: shear wave elastography, SD: standard deviation, CI: confidence interval, kPa: kilopascal

Parameter	HDP group (n = 18), mean ± SD	Non-HDP group (n = 57), mean ± SD	Mean difference (95% CI)	t-value	p-value
EMAX Average (kPa)	13.09 ± 4.79	10.24 ± 4.02	2.85 (0.58 to 5.12)	2.504	0.015
EMEAN Average (kPa)	7.33 ± 2.88	5.93 ± 2.07	1.40 (0.17 to 2.63)	2.261	0.027
EMIN Average (kPa)	4.11 ± 1.62	3.47 ± 1.38	0.64 (-0.14 to 1.42)	1.643	0.105

**Figure 5:**
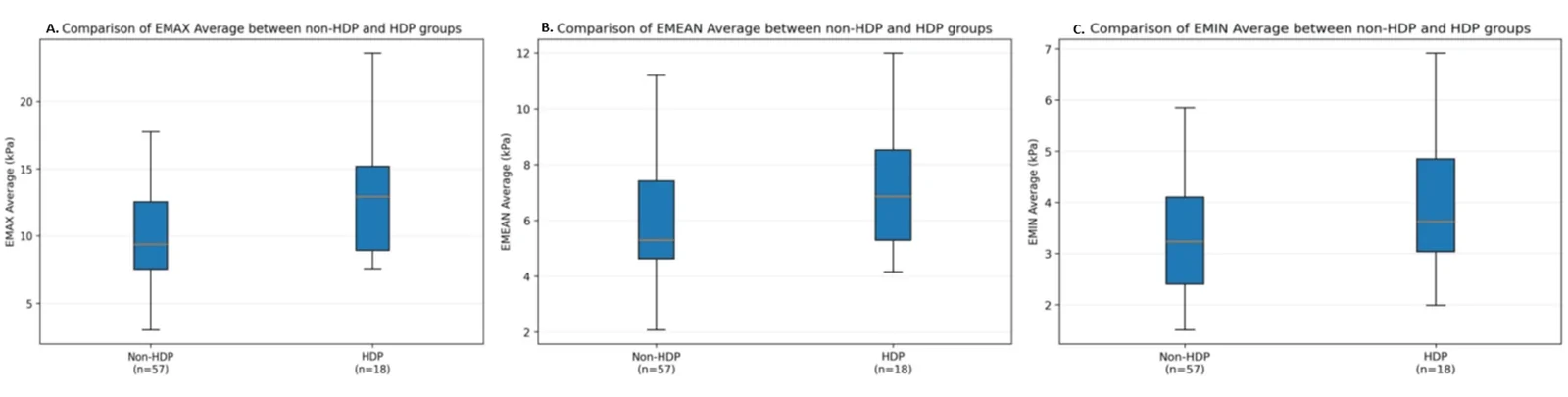
Box plots showing the distribution of placental SWE parameters between non-HDP and HDP groups. A) EMAX Average; B) EMEAN Average; C) EMIN Average. The central line represents the median, the box represents the interquartile range, and the whiskers represent the range. HDP: hypertensive disorders of pregnancy; SWE: shear wave elastography; kPa: kilopascal

The distribution of the averaged placental SWE parameters between the non-HDP and HDP groups is shown in Figure 5. EMAX Average and EMEAN Average showed higher values in the HDP group, while EMIN Average showed greater overlap between groups.

ROC analysis

ROC curve analysis was performed for EMAX Average, EMEAN Average, and EMIN Average. The ROC findings are summarized in Table 3.

**Table 3:** ROC analysis of average placental SWE parameters for prediction of HDP. AUC: area under the curve, CI: confidence interval, HDP: hypertensive disorders of pregnancy, ROC: receiver operating characteristic, SWE: shear wave elastography, kPa: kilopascal

Parameter	AUC (95% CI)	p-value	Cut-off	Sensitivity	Specificity
EMAX Average	0.682 (0.564-0.785)	0.009	>11.71 kPa	61.1%	73.7%
EMEAN Average	0.643 (0.524-0.750)	0.058	>5.41 kPa	72.2%	57.9%
EMIN Average	0.607 (0.488-0.718)	0.157	>2.84 kPa	88.9%	40.4%

**Figure 6:**
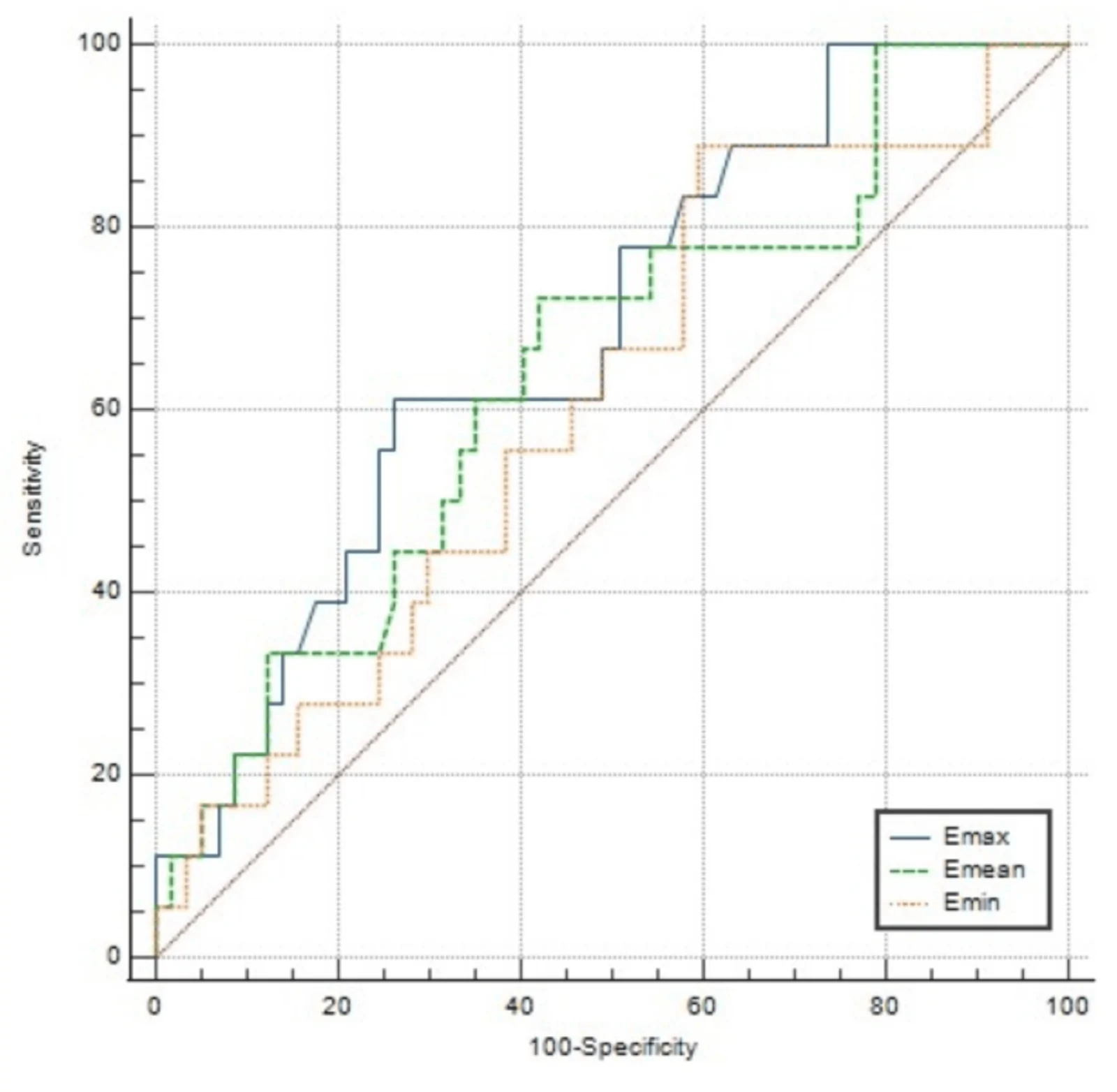
Combined ROC curve of EMAX Average, EMEAN Average, and EMIN Average for prediction of HDP. ROC: receiver operating characteristic; HDP: hypertensive disorders of pregnancy

The combined ROC curve of EMAX Average, EMEAN Average, and EMIN Average is shown in Figure 6. The EMAX Average curve showed the most useful overall discrimination among the three averaged elastography parameters.

EMAX Average showed the best diagnostic performance, with an area under the curve of 0.682 (95% CI: 0.564-0.785; p = 0.009). At the exploratory Youden-derived cut-off value of >11.71 kPa, sensitivity was 61.1% and specificity was 73.7%. The positive likelihood ratio was 2.32 and the negative likelihood ratio was 0.53. The positive predictive value was 42.3% and the negative predictive value was 85.7%. EMEAN Average showed an area under the curve of 0.643 (95% CI: 0.524-0.750; p = 0.058). At the cut-off value of >5.41 kPa, sensitivity was 72.2% and specificity was 57.9%. EMIN Average showed an area under the curve of 0.607 (95% CI: 0.488-0.718; p = 0.157). At the cut-off value of >2.84 kPa, sensitivity was 88.9% and specificity was 40.4%. Pairwise comparison of ROC curves did not show statistically significant differences between the three averaged elastography parameters.

Baseline comparison and follow-up blood pressure trends

Baseline maternal age and USG-AUA-based gestational age were compared between the HDP and non-HDP groups, as presented in Table 4. Neither variable showed a statistically significant difference between groups at the time of first-trimester SWE assessment.

**Table 4:** Baseline comparison between HDP and non-HDP groups. Values are expressed as mean ± standard deviation. Mean difference = HDP group - non-HDP group. Independent sample t-test was used. HDP: hypertensive disorders of pregnancy; USG-AUA: ultrasound-based average ultrasound age; CI: confidence interval; SD: standard deviation

Variable	HDP group (n=18), mean ± SD	Non-HDP group (n=57), mean ± SD	Mean difference (95% CI)	t-value	p-value
Maternal age (years)	31.33 ± 4.35	29.18 ± 3.91	2.16 (-0.01 to 4.32)	1.988	0.051
USG-AUA-based gestational age (weeks)	12.75 ± 1.01	12.54 ± 0.82	0.20 (-0.27 to 0.67)	0.862	0.391

**Figure 7:**
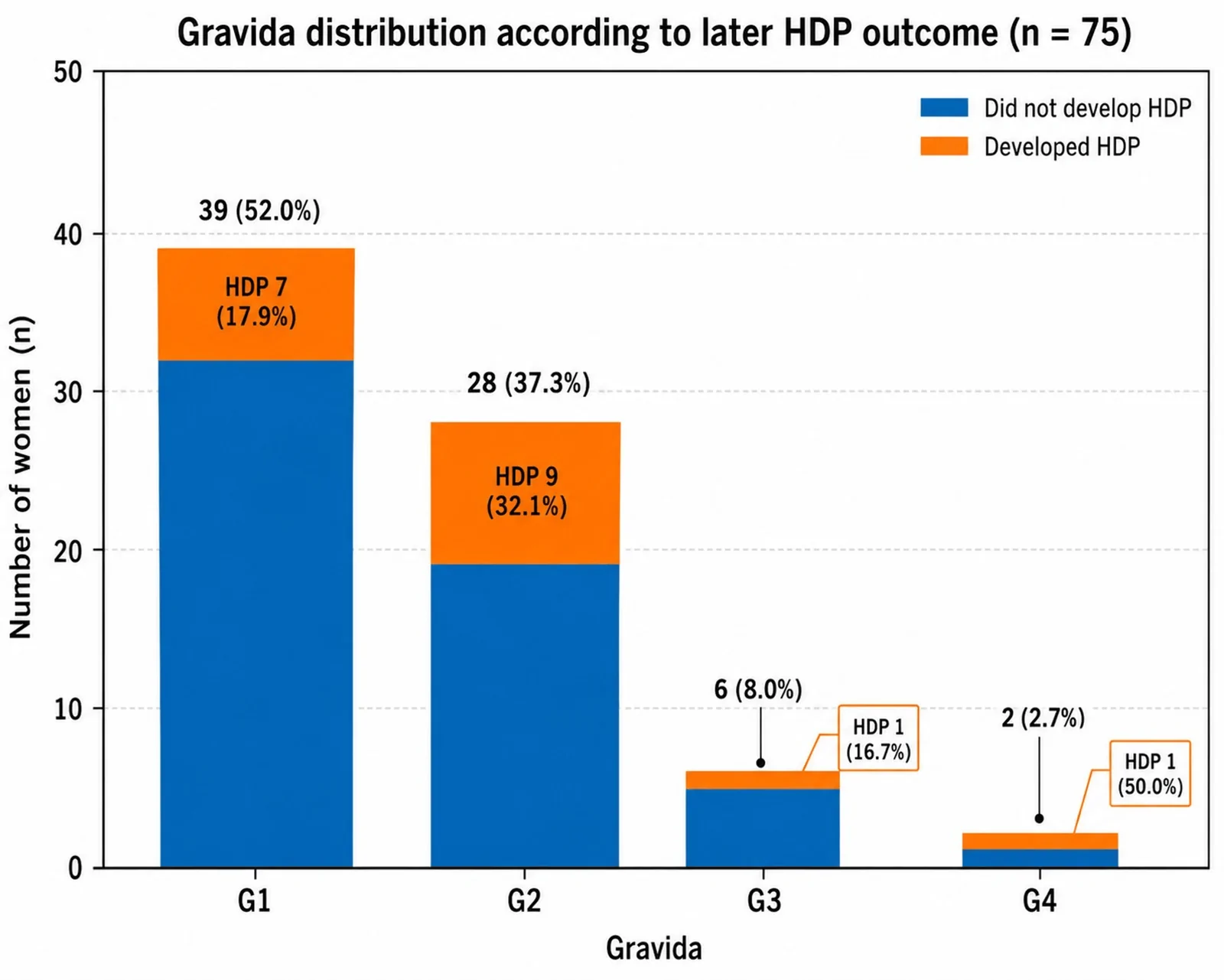
Stacked bar chart showing gravida distribution according to later HDP outcome. The total height of each bar represents the number of women in each gravida category, while the highlighted segment represents women who later developed HDP. HDP: hypertensive disorders of pregnancy; G: gravida

Gravida distribution according to later HDP outcome is shown in Figure 7. The figure presents the total number of women in each gravida category and the proportion who later developed HDP.

**Figure 8:**
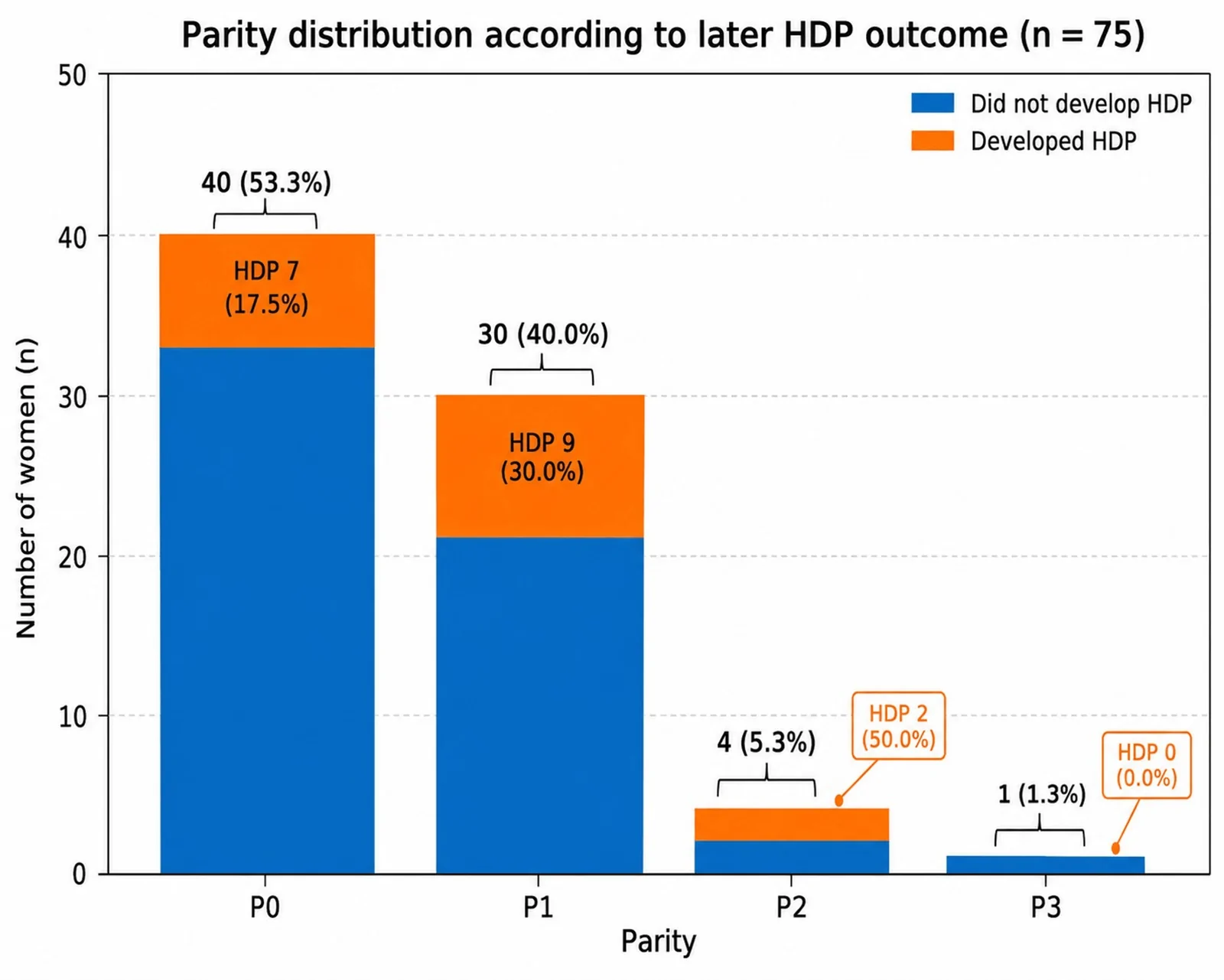
Stacked bar chart showing parity distribution according to later HDP outcome. The total height of each bar represents the number of women in each parity category, while the highlighted segment represents women who later developed HDP. HDP: hypertensive disorders of pregnancy; P: parity

Parity distribution according to the later HDP outcome is shown in Figure 8. In the parity distribution, HDP occurred in 7 of 40 P0 women, 9 of 30 P1 women, and 2 of 4 P2 women; no woman in the P3 category developed HDP.

**Figure 9:**
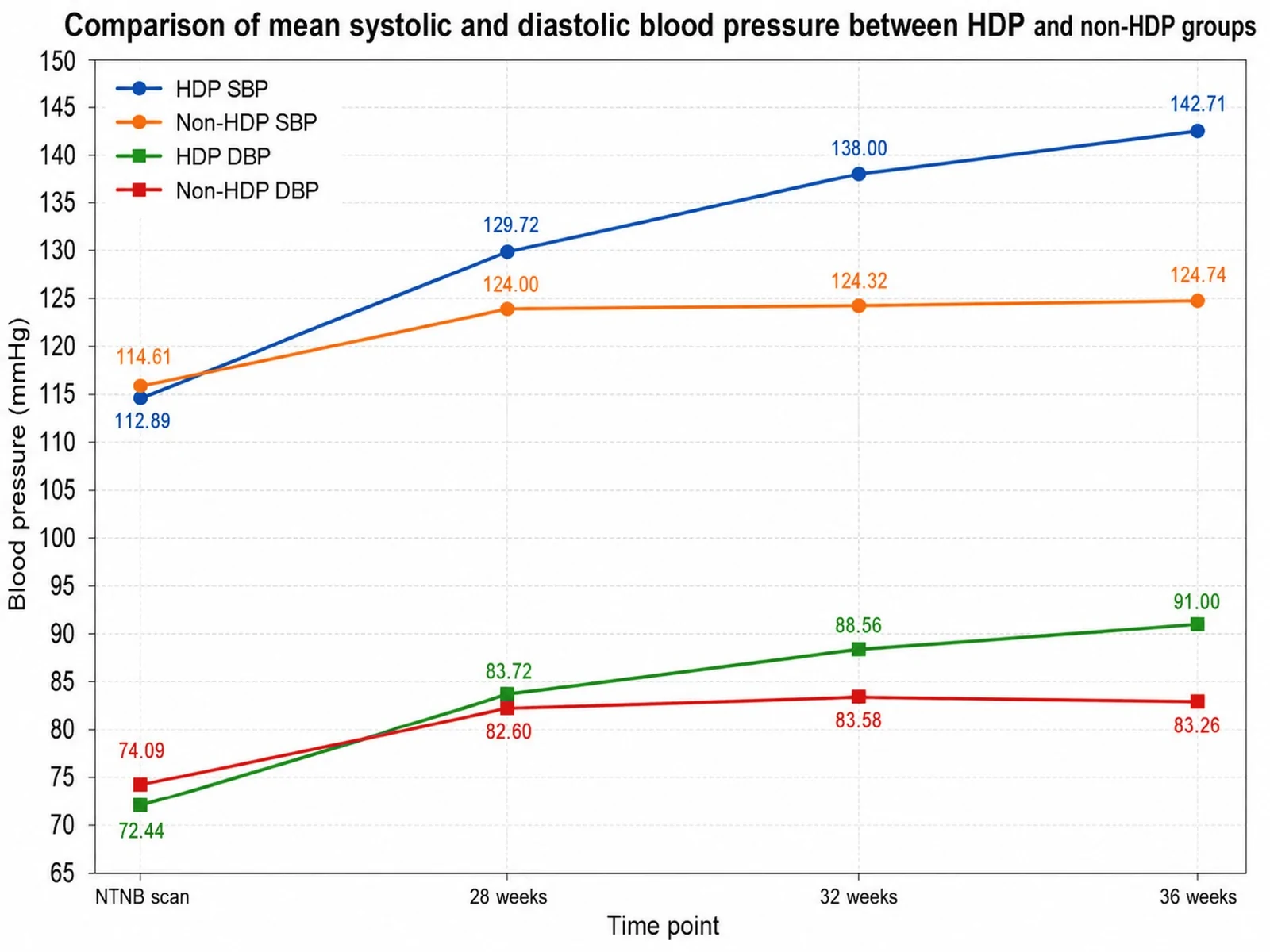
Line graph showing comparison of mean systolic and diastolic blood pressure between HDP and non-HDP groups during follow-up. HDP: hypertensive disorders of pregnancy; SBP: systolic blood pressure; DBP: diastolic blood pressure; NTNB: nuchal translucency and nasal bone scan

At the NTNB scan, systolic and diastolic blood pressure values did not show statistically significant differences between the HDP and non-HDP groups. The later follow-up trend is shown in Figure 9. During follow-up, systolic blood pressure became significantly higher in the HDP group at 28, 32, and 36 weeks, while diastolic blood pressure was significantly higher at 32 and 36 weeks. This confirmed that first-trimester placental SWE measurements were obtained before the later clinical separation of blood pressure values.

Other associated clinical conditions and delivery outcome

Other associated medical and obstetric conditions were reviewed descriptively. Recorded medical conditions included hypothyroidism/overt hypothyroidism, gestational diabetes mellitus, and intrahepatic cholestasis of pregnancy. Some women had more than one associated medical condition. Among the non-HDP obstetric conditions, premature rupture of membranes was the most frequent. Oligohydramnios was also noted in a small number of women. These associated clinical conditions were documented to describe the background clinical profile of the cohort and were not used as independent predictors in the primary elastography analysis.

Gestational age at delivery in the HDP group was lower than that in the non-HDP group (37.89 ± 1.49 weeks vs 38.72 ± 0.92 weeks; p = 0.006). This finding was reviewed as an associated follow-up outcome and not as the primary predictive endpoint.

## Discussion

This prospective hospital-based study evaluated the association and diagnostic performance of placental SWE performed during the 11- to 14-week scan for later development of HDP. The final analysis included 75 women, of whom 18 developed HDP. The main finding was that EMAX Average and EMEAN Average were significantly higher in women who later developed HDP, while EMIN Average did not show a statistically significant difference. Among the three averaged elastography parameters, EMAX Average showed the best receiver operating characteristic performance.

The finding of increased placental stiffness in the HDP group is biologically plausible. Abnormal placentation, defective spiral artery remodeling, and placental malperfusion are important mechanisms in preeclampsia and related hypertensive disorders [7-9]. Placental hypoperfusion and microstructural alteration may contribute to changes in tissue stiffness. SWE provides a quantitative method for assessing this biomechanical property [12,13]. However, the present study did not include placental histopathological correlation, so the precise tissue correlate of increased stiffness could not be confirmed.

The present findings are broadly consistent with earlier placental elastography studies. Kılıç et al. evaluated placental SWE in the second and third trimesters and found higher placental stiffness in preeclamptic pregnancies than in controls [14]. Meena et al. reported higher placental stiffness between 16 and 20 weeks in women who later developed preeclampsia [15]. Singh et al. reported recent Indian second-trimester data from a high-risk cohort, with higher placental SWE values in women who developed preeclampsia [17]. These studies support the association between increased placental stiffness and hypertensive placental disease.

Sirinoglu et al. is one of the most directly comparable studies because it evaluated first-trimester placental SWE between 11 and 13 weeks + 6 days. They included 84 women, of whom nine developed preeclampsia, and assessed mean placental SWE values from multiple placental areas [18]. In comparison, the present study included 75 women, with 18 developing the broader HDP outcome, and analyzed averaged EMAX, EMEAN, and EMIN values. Sirinoglu et al. reported a cut-off of 7.43 kPa with sensitivity and specificity of 88% and 78%, respectively [18]. In comparison, the present study showed more modest diagnostic performance, with EMAX Average showing an AUC of 0.682, sensitivity of 61.1%, and specificity of 73.7%. Differences in outcome definition, study population, ultrasound platform, region-of-interest methodology, placental region sampled, number of affected cases, and elastographic parameter analyzed may explain this variation. The present study used a broader HDP outcome that included pregnancy-induced hypertension/gestational hypertension and preeclampsia, whereas some previous studies focused primarily on preeclampsia.

EMAX Average appeared more useful than EMEAN Average and EMIN Average. EMAX reflects the highest stiffness component within the sampled placental region. If placental malperfusion or tissue alteration is patchy, maximum stiffness may capture focal abnormality better than mean or minimum values. EMEAN Average may be influenced by both abnormal and relatively preserved tissue, while EMIN Average may reflect softer preserved regions. This explanation should be interpreted cautiously because histopathological confirmation was not performed.

An important finding was the difference between group comparison and diagnostic classification. EMEAN Average was significantly higher in the HDP group on t-test, but its receiver operating characteristic performance did not reach statistical significance. This suggests that although EMEAN Average differed between groups, its ability to classify individual women as HDP or non-HDP was limited. This distinction is important in diagnostic research. A statistically significant mean difference does not necessarily imply strong clinical usefulness or reliable individual-level prediction.

The moderate AUC of EMAX Average indicates that first-trimester placental SWE should not be interpreted as a standalone screening tool. The Youden-derived cut-off of >11.71 kPa should be considered exploratory and internally derived rather than a clinically validated threshold. Current first-trimester screening performs best when multiple variables are combined, including maternal factors, blood pressure or mean arterial pressure, uterine artery Doppler, and biochemical markers [10,11]. Tian et al. also reported that combining placental SWE with Doppler-related parameters improved prediction of preeclampsia [19]. In this context, SWE may be more useful as part of a multiparametric model rather than as an isolated marker. In the present study, multivariable logistic regression was not performed because only 18 HDP outcome events occurred, and inclusion of multiple predictors would risk overfitting and unstable estimates. Therefore, the present findings demonstrate univariable association and exploratory diagnostic performance, but do not establish SWE as an independent predictor of HDP.

Baseline systolic and diastolic blood pressure values at the NTNB scan were not significantly different between women who later developed HDP and those who remained normotensive. This supports the temporal relevance of the study design, because placental SWE was performed before clinical blood pressure separation became evident. During follow-up, systolic blood pressure became significantly higher in the HDP group at 28, 32, and 36 weeks, and diastolic blood pressure became significantly higher at 32 and 36 weeks. This confirms that the outcome classification was based on the later clinical development of hypertensive disease.

The study has several strengths. It was prospective in design and performed during the first-trimester NTNB scan window. Averaged values from three placental SWE measurements were used instead of relying on a single region of interest. Follow-up was performed prospectively, allowing correlation between first-trimester placental stiffness and later clinical outcomes. The examinations were performed by four radiologists with experience ranging from 2 to 17 years, from Senior Resident to Professor level, using a standardized acquisition protocol. Operators were blinded to maternal risk factors and clinical history at the time of SWE acquisition, and outcome assessors were blinded to SWE values. Measurements were accepted only when the machine-generated M-STB stability indicator showed five stars, improving technical consistency. The study also provides region-specific data from North-East India, where published first-trimester placental elastography data are limited.

The study also has important limitations. Although 80 women were initially enrolled based on the calculated sample size, the final analyzed cohort included 75 women after losses to follow-up and abortion, which was slightly below the calculated minimum sample size of 79. Only 18 women developed HDP, limiting the stability of ROC estimates, subgroup analysis, and cut-off derivation. The internally derived EMAX Average cut-off was exploratory and was not externally validated. The study was conducted at a single tertiary care hospital, which may limit generalizability. Although SWE was performed by trained radiologists using a standardized protocol and five-star M-STB stability readings, formal interobserver and intraobserver reproducibility assessment was not performed. This is an important limitation because SWE measurements may be influenced by operator technique, placental depth, fetal movement, maternal breathing, body habitus, placental location, transducer pressure, and region-of-interest placement.

Another limitation is that maternal BMI, uterine artery Doppler, mean arterial pressure, biochemical markers such as PlGF or PAPP-A, aspirin prophylaxis, and other established screening variables were not included in the primary model. Therefore, the independent or incremental predictive value of SWE over established first-trimester screening methods could not be assessed. Outcome classification was based on antenatal and delivery clinical records, and proteinuria, maternal end-organ dysfunction, and uteroplacental dysfunction were assessed from clinical documentation. Thermal index, mechanical index, and exact SWE exposure duration were not separately recorded, although ultrasound scanning was performed following the ALARA principle. Placental histopathological correlation was also not performed, so the tissue basis of increased stiffness could not be confirmed.

Future studies should evaluate first-trimester placental SWE in larger multicentric cohorts with adequate outcome events, formal interobserver and intraobserver reproducibility assessment, standardized acquisition protocols, and external validation of cut-off values. Further research should also assess whether placental SWE improves established screening models when combined with maternal risk factors, mean arterial pressure, uterine artery Doppler, and biochemical markers.

**Figure 10:**
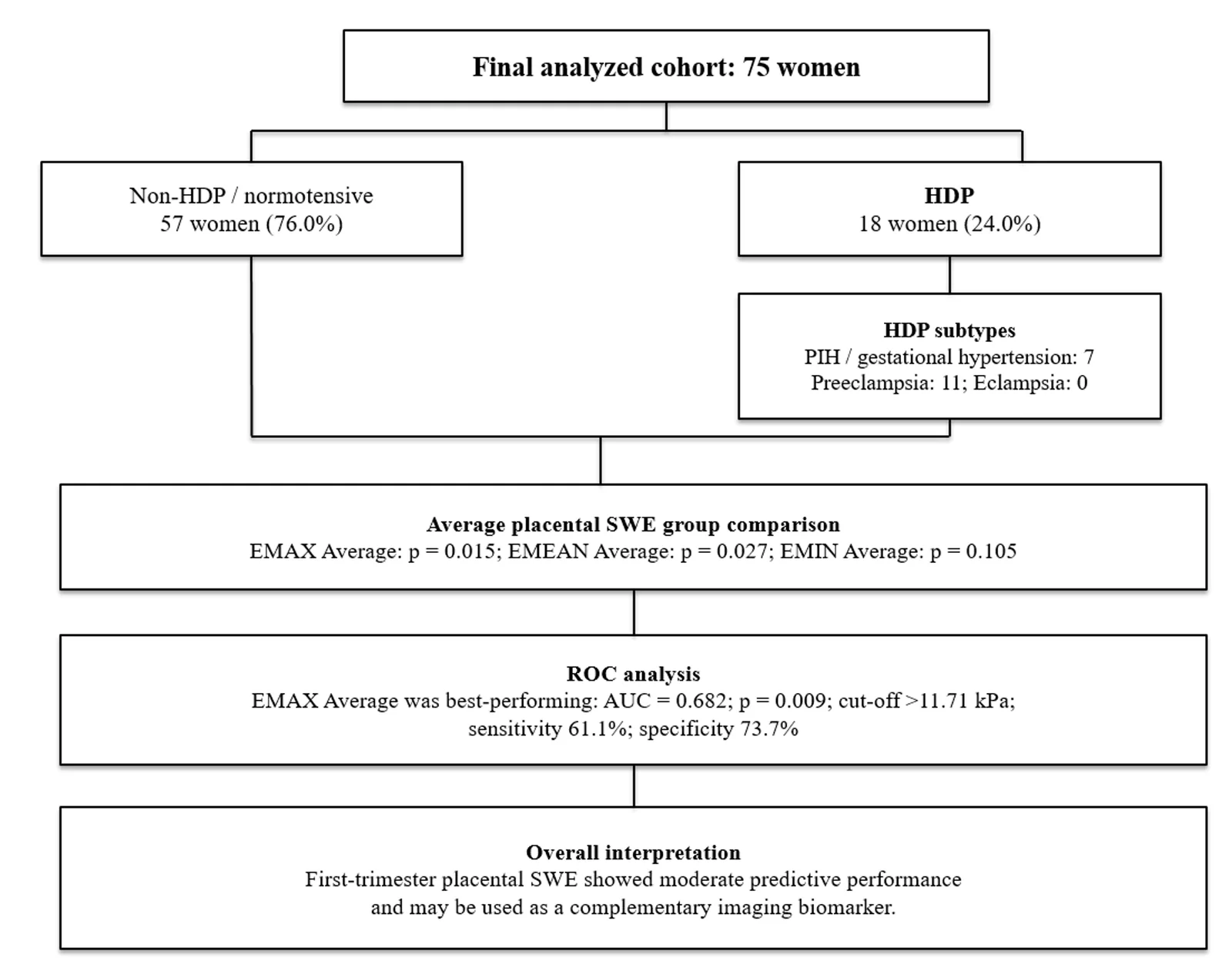
Flowchart summarizing the main results of the study. ROC: receiver operating characteristic; HDP: hypertensive disorders of pregnancy; SWE: shear wave elastography

Figure 10 summarizes the main result of the study.

## Conclusions

First-trimester placental SWE performed during the routine 11- to 14-week ultrasound scan was feasible and provided quantitative placental stiffness measurements before the clinical development of HDP. EMAX Average and EMEAN Average were significantly higher in women who later developed HDP compared with women who remained normotensive, while EMIN Average showed limited discriminatory value. Among the evaluated parameters, EMAX Average showed the best diagnostic performance, with an AUC of 0.682 and an exploratory cut-off value of >11.71 kPa; however, sensitivity and specificity were moderate.

These findings suggest that first-trimester placental SWE, particularly EMAX Average, may serve as a complementary imaging biomarker for later HDP rather than a standalone screening test. Larger multicentric studies with standardized acquisition protocols, formal reproducibility assessment, external validation of cut-off values, and integration with established screening markers are required before routine clinical implementation.
